# The Health Benefiting Mechanisms of Virgin Olive Oil Phenolic Compounds

**DOI:** 10.3390/molecules21121734

**Published:** 2016-12-16

**Authors:** Lisa Parkinson, Sara Cicerale

**Affiliations:** 1 Department of Medical and Health Science, Swinburne University, Hawthorn, VIC 3122, Australia; 2 School of Exercise and Nutrition Sciences, Centre for Advanced Sensory Science (CASS), Deakin University, Burwood, VIC 3125, Australia; sara.cicerale@deakin.edu.au

**Keywords:** olive oil phenolics, olive oil, health benefits, inflammation

## Abstract

Virgin olive oil (VOO) is credited as being one of the many healthful components associated with the Mediterranean diet. Mediterranean populations experience reduced incidence of chronic inflammatory disease states and VOO is readily consumed as part of an everyday Mediterranean dietary pattern. VOO is rich in phenolic compounds and the health promoting benefits of these phenolics are now established. Recent studies have highlighted the biological properties of VOO phenolic compounds elucidating their anti-inflammatory activities. This paper will review current knowledge on the anti-inflammatory and nutrigenomic, chemoprotective and anti-atherosclerotic activities of VOO phenolics. In addition the concentration, metabolism and bioavailability of specific phenolic compounds will be discussed. The evidence presented in the review concludes that oleurepein, hydroxytyrosol and oleocanthal have potent pharmacological activities in vitro and in vivo; however, intervention studies with biologically relevant concentrations of these phenolic compounds are required.

## 1. Introduction

The health promoting attributes associated with following a traditional Mediterranean diet have been reviewed extensively. The first suggestion of healthful effects arose from the Seven Countries Study conducted in the 1970’s [1]. It has since been confirmed that the prevalence of chronic inflammatory disease for Mediterranean populations is the lowest in the world, and life expectancy amongst the highest [2]. As a result, nutrition research has focused on populations residing along the Mediterranean Sea to uncover key nutrients and dietary patterns that may be associated with reported superior health status. Since the early Seven Countries Study, several other studies have supported the view that this pattern of eating is associated with a reduced incidence of inflammatory disease states [3,4,5,6,7,8,9,10,11,12,13,14,15,16,17]. This review discusses the modes of action through which VOO phenolic compounds may exert their health protective effects, together with information on the concentrations in which they are found in VOO, their degree of bioavailability and metabolism. A search for relevant literature published in English was conducted via PubMed. Relevant research articles were identified using the following key words: olive oil phenolics, hydroxytyrosol, oleuropein, oleocanthal and health benefits, inflammation, inflammatory disease, cancer, obesity, Alzheimer’s disease, bioavailability. The reference lists of included articles and recent significant reviews were also examined to identify relevant publications. 

## 2. Virgin Olive Oil

The principle source of dietary fat in the Mediterranean diet is VOO [18,19] and VOO contributes significantly to the superior health profile observed in Mediterranean populations [20,21]. In fact, throughout history, VOO has been recognized as being a potent pharmacological agent by ancient Greek doctors, and Hippocrates mentions approximately 60 health conditions where VOO use can be beneficial [22]. A large body of evidence has highlighted the benefits of VOO intake on primary end points for cardiovascular disease (CVD) and total mortality as well as secondary risk factors for chronic inflammatory diseases such as cancer, endothelial function and hypertension [23]. 

## 3. Phenolic Fraction

It is now known that VOO contains approximately 36 phenolic compounds and these compounds play a key role in the health promoting qualities of the oil [7,23,24,25,26]. Research investigating VOO phenolics and benefits to human health has focused on inflammation, antioxidant status, antimicrobial activity, as well as other biological markers of non-communicable disease (for an earlier review see [25]). The principle phenolics of interest in this review are secoiridoid oleuropein, and its derivative hydroxytyrosol, and the minor phenolic compound oleocanthal.

## 4. Virgin Olive Oil Phenolic Concentration 

Over 30 VOO phenolic compounds have been identified and considerable variation has been noted in the concentration of such phenolic compounds (0.02 to 600 mg/kg) [24]. VOO phenolic compounds can be grouped into the following: phenolic acids, phenolic alcohols, secoiridoids, hydroxy-isocromans, flavonoids and lignans. Phenolic acids are present in the smallest of quantities in VOO. Conversely, secoiridoids are present in the largest of quantities in VOO. Phenolic alcohols, flavonoids and lignans are present in quantities between those of phenolic acids and secoiridoids. Quantity data for hydroxy-isocromans are currently limited. 

A number of factors have been shown to affect the concentration of phenolic compounds in VOO and these include olive fruit cultivar and variety, region of growth, agricultural cultivation techniques, maturity at harvest, processing, and storage. Also, olive tree age has been shown to influence phenolic content [24]. 

## 5. Virgin Olive Oil Bioavailability and Metabolism 

The phenolic concentration and composition of VOO together with the degree to which these components are absorbed and metabolized are essential in determining the health effects associated with such compounds. Bioavailability of VOO phenolic compounds is the key in achieving an effect in specific tissues or organs [27].

The majority of research regarding the bioavailability of these compounds has focused on the two most abundant VOO phenolics: hydroxytyrosol and tyrosol, amongst a few others [28]. Research evidence demonstrates that there is significant absorption (~40%–95%) of these compounds in a dose-dependent manner in humans [29,30,31,32,33]. Moreover, Tuck et al. [32] noted the increase in bioavailability of hydroxytyrosol and tyrosol when administered as an olive oil solution compared to an aqueous solution. This finding suggests the VOO itself may have acted as a protective factor preventing the breakdown of the phenolics in the gastrointestinal tract prior to absorption [32]. Vissers and colleagues [31] found that absorption of administered ligstroside-aglycone, hydroxytyrosol, tyrosol, and oleuropein-aglycone was 55%–66% in human subjects. A much smaller quantity of these phenolics was recovered in urine (5%–16%). Furthermore, Miro Casas and colleagues [33] demonstrated a mean urinary recovery of 25% of administered tyrosol. Visioli et al. [34] also reported urinary excretion of administered hydroxytyrosol as 30%–60% and 20%–22% for tyrosol. These figures indicate that humans absorb a major part of VOO phenolics ingested.

With regards to the metabolism of VOO phenolics, the presence of metabolites from the majority of olive oil phenolic compounds (i.e., secoiridoids, flavanoids and phenolic alcohols) in human urine has been noted [35]. This implies that such compounds are metabolized and absorbed post-ingestion. Varying rates of metabolism amongst differing VOO phenolics have also been found. The largest number of metabolites produced post-ingestion have been found with hydroxytyrosol, oleuropein aglycone and oleocanthal, with circulating metabolites ranging from 10–60 μg post-ingestion of a 50 mL high phenol-containing VOO. By contrast, the lowest number of metabolites have been found with tyrosol, luteolin, apigenin, pinoresinol and acetoxypinoresinol (concentration not stated in study) [35]. In the case of poorly absorbed VOO phenolics, it has been postulated that these compounds may exert local antioxidant activities in the gastrointestinal tract and this proposal is supported by research demonstrating the free radical scavenging capacity of VOO phenolics in both the fecal matrix and intestinal epithelial cells [36]. Further, it is has been suggested that unabsorbed VOO phenolics may exert antimicrobial activities in the gastrointestinal tract [37]. 

VOO intake for Mediterranean populations is reported to be 30–50 g/day [38]. If we assume this quantity of VOO contains 200 μg phenolics and the absorption rate is in the range of 40%–95% of the ingested olive oil phenols, it may be assumed that the quantity of ingested olive oil phenols are in the range of 4–9 mg/day. While the in vivo studies discussed in this review suggest physiologically relevant concentrations, many in vivo studies use supraphysiological concentrations (>10 μM) [39]. Therefore, it is difficult to translate this into physiological relevance, and future research is needed using concentrations that can be extrapolated to humans. Also of consideration is that VOO phenolics are consumed in a diet and possibly interact with other compounds within the food matrix. Therefore, it is important to consider such factors when investigating the health benefits of such components in humans.

## 6. Virgin Olive Oil Phenolics as Anti-Inflammatory and Nutraceutical Agents 

It is known that the pathophysiology of common disease states such as cancer, CVD, arthritis and neurodegenerative disease takes root in chronic inflammation [40]. Phenolic compounds derived from VOO have been reported to possess significant anti-inflammatory qualities. It has been reported that postprandial inflammatory response after ingestion of heated vegetable oils in obese individuals is significantly reduced by phenolic-rich VOO [41]. Although the effect of monounsaturated fatty acids in VOO may be similar as that of the phenolic component, the authors report that VOO or a mix of sunflower and rapeseed oil artificially enriched with olive oil phenolic compounds and other antioxidants mitigate postprandial inflammation. The phenolic enriched oil reduces nuclear factor kappa-light-chain-enhancer of activated B cells (NF-κB) activation, increases nuclear factor of kappa light polypeptide gene enhancer in B-cells inhibitor, alpha (IκB-a), and decreases lipopolysaccharide (LPS) plasma concentration, compared with sunflower oil, demonstrating the potent effects that VOO phenolics have on inflammatory markers. 

There are now several studies that have examined the anti-inflammatory and cardio-protective effects of olive oil phenolic compounds in humans. These studies suggest that VOO with high phenolic concentration is effective in modulating inflammatory mediators derived from arachidonic acid, such as thromboxane B2 and 6-keto-PG F1a, as well as other inflammatory markers, such as high-sensitivity C-reactive protein (CRP) and interleukin 6 (IL-6) [42,43,44]. More recently, Muto and colleagues [45] investigated the effects of olive oil phenolics on Caco-2 cells exposed to the inflammatory effects of LPS. They report that an olive oil phenolic extract attenuates interleukin 8 (IL-8) expression and that the phenolic fraction can modulate an acute inflammatory response in intestinal epithelial cells. Table 1 lists the in vitro and in vivo studies demonstrating the effect of oleocanthal, oleuropein, and hydroxytyrosol on markers of disease.

In regard to the specific actions of the phenolic oleuropein, early evidence demonstrates this phenolic inhibits tumour necrosis factor alpha (TNFa) induced matrix metalloproteinase 9 (MMP-9) in a monocyte cell line. This has implications for health as monocytes, and the molecules they secrete play a significant role in inflammatory disease development [46]. Further, the administration of oleuropein 30 min after challenge with TNFa produced a significant reduction in cytokine induced matrix metalloproteinases (MMPs), which amplify the inflammatory response and also contribute to atherosclerotic changes in arterial walls [46].

Furthermore, the phenolic compound oleocanthal has been found to share the same mechanistic anti-inflammatory pathway as the non-steroidal anti-inflammatory drug, ibuprofen. In vitro, oleocanthal has been shown to inhibit both cyclooxygenase-1 (COX-1) and cyclooxygenase-2 (COX-2) inflammatory enzymes in a dose-dependent manner, and is more effective than ibuprofen in inhibiting these enzymes at equimolar concentrations [47]. The anti-inflammatory actions of oleocanthal extend to pharmacological actions in attenuating inflammatory mediators such as inducible nitric oxide synthase (iNOS) which plays a role in the pathogenesis of joint degenerative disease [48,49]. 

Further evidence suggests that oleocanthal may be a potent pharmacological agent in the treatment of neurogenerative disease, as this compound exhibits neuroprotective properties, in addition to attenuating markers of inflammation implicated in Alzheimer’s disease [50,51,52,53]. However, it must be considered that results from in vitro models may not extend to in vivo models of disease due to the inability of some compounds to cross the blood-brain barrier. Abuznait and colleagues (2015) report that expression of P-glycoprotein (P-gp) and LDL lipoprotein receptor related protein-1 (LRP1) in the brain microvessels of C57BL/6 mice is increased after oleocanthal treatment These transport proteins are responsible for the clearance of β-amyloid (Aβ) across the blood-brain barrier and Aβ is a hallmark in the risk of Alzheimer’s disease. Therefore, it would seem that oleocanthal induces P-gp and LRP1 and enhances Aβ clearance across the blood brain barrier [53,54].

Hydroxytyrosol has been reported to possess significant anti-inflammatory actions in an animal model of inflammation and to attenuate TNFa and interleukin 1β (IL-1b) expression, which are pro-inflammatory cytokines often observed in inflammatory disease [55] Furthermore, in vitro evidence shows the attenuation of pro-inflammatory agents iNOS, COX-2 and TNFa by hydroxytyrosol in LPS-challenged human monocytic THP-1 cells [56]. 

## 7. Virgin Olive Oil Phenolics and Cancer

Due to the anti-inflammatory efficacy of VOO phenolics, much research has focused on the pharmacological properties of these compounds, in particular the anti-carcinogenic actions. There is much research suggesting that oleuropein and its metabolite, hydroxytyrosol, exert anti-cancer properties. An early study has shown that high doses of oleuropein at a concentration of 200 μg/mL decreased cell viability and inhibited cell proliferation in human breast adenocarcinoma (MCF-7) breast cancer cells [58]. Furthermore, a study using oleuropein as the dominant compound from crude extracts derived from the olive leaf inhibited cell proliferation of MCF-7, human urinary bladder carcinoma and bovine brain capillary endothelial [71]. Of importance is an in vivo study [70] that showed oleuropein possesses anti-cancer activity and inhibited both MCF-7 cell growth and their invasiveness into the lung. This study demonstrates that oleuropein prevents both peripulmonary and parenchyma lung metastases in vivo [70]. 

Chimento and colleagues [59] report that micromolar concentrations of oleupopein and hydroxytyrosol reduce SKBR3 cell growth by exhibiting sustained ERK1/2 activation, triggering an intrinsic apoptotic pathway. The VOO phenolic oleocanthal has also been shown to possess anti-proliferative effects in human breast, prostate, and bone cancer lines [60,61]. Recently, LeGendre and colleagues [62] reported that oleocanthal promoted primary necrotic cell death in serum-starved cancer cells. This was associated with elevated levels of phosphorylated ERK1/2 in the absence of cleaved caspase-3 expression. In serum, the cancer cells exhibited both apoptosis and secondary necrosis. Additionally, oleocanthal has been shown to inhibit proliferation and induced apoptosis in hepatocellular carcinoma (HCC) cells in vitro [69]. It was reported that the oleocanthal treated group of mice had fewer and smaller lung metastases compared to the control group [69]. The study concluded that oleocanthal inhibits HCC tumor growth and metastasis by inactivating STAT3 both in vitro and in vivo. STAT3 is a transcription factor and is implicated in the survival, proliferation, invasion, and angiogenesis of HCC by regulating the expression of target genes. Therefore, STAT3 may be a promising target for HCC therapy [69]. A recent study has also shown that oleocanthal inhibits extracellular signal-regulated kinases 1/2 (ERK1/2) and AKT phosphorylation as well as down-regulates Bcl-2 expression. AKT, ERK1/2 and are necessary, not only for c-Met-mediated regulation of cell motility, adhesion, and invasion, but also for control of cell survival and mitogenesis [72] and through this, induces cytotoxicity against human melanoma cells [63].

A recent study showed that oleocanthal inhibits the mammalian target of rapamycin (mTOR) [64]. mTOR is a serine/threonine kinase and member of the PI3K-related kinase family. It has a crucial role in integrating signals from energy homeostasis, metabolism, stress response, and the cell cycle, and dysregulated PI3K/mTOR signaling is frequently observed in cancers [73]. Oleocanthal treatment (10 μM) caused a marked downregulation of the phosphorylated mTOR (p-mTOR) in a metastatic breast cancer cell line (MDA-MB-231), suggesting that the anti-carcinogenic activity of oleocanthal is potentially mediated by mTOR inhibition [64]. 

## 8. Virgin Olive Oil Phenolics and Atherosclerosis

Early research has shown that VOO phenolics improve endothelial dysfunction and reduce oxidative stress plasma parameters [74,75], which play a key role in the development of atherosclerosis. In support of the beneficial effects VOO phenolics exert on atherosclerosis risk factors, Konstantinidou and colleagues [76] reported that VOO containing 328 mg/kg compared to 55 mg/kg of phenolics down-regulates inflammatory genes Rho-GTPase-activating protein 15, interferon gamma and interleukin 7 receptor and also adrenergic beta 2 receptor and polymerase kappa; these are all implicated in atherosclerosis. Using a rabbit animal model, Gonzalez and colleagues [68] reported that in the presence of saturated fat and cholesterol, hydroxytyrosol at 4 mg/kg reduced the size of atherosclerotic lesions when compared with rabbits receiving this diet without hydroxytyrosol [68]. 

Furthermore, a recent randomized control trial reported that VOO phenolic intake decreased low density lipoprotein (LDL) concentrations and LDL atherogenicity in vivo [77]. This study involved the intake of 25 mL/day of raw high-polyphenol content olive oil (366 mg/kg) or low-polyphenol-content olive oil (2.7 mg/kg) for 3 weeks. VOO phenolics decreased LDL concentrations directly measured as concentrations of apo B-100 and the total number of LDL particles. Additionally, conclusions were drawn that VOO phenolics decrease LDL atherogenicity, which was reflected in the lower number of small LDL particles and enhanced LDL resistance to oxidation [77]. These studies add to previous evidence that VOO phenolics improve oxidative status and reduce cardiovascular risk factors [75,78,79]. 

## 9. Virgin Olive Oil Phenolics and Obesity

VOO phenolics have been shown to influence the expression of genes related to obesity. Warnke and colleagues observed that hydroxytyrosol at a concentration of 25 μM was able to modify genes related to adipocyte maturation and differentiation and to have an inhibitory effect on lipid formation [65]. Hyperlipidemia is detrimental to coronary arteries and the most important risk factor for heart disease. Additionally, Drira and colleagues reported that both hydroxytyrosol and oleuropein at concentrations of 100 and 150 μM and 200 and 300 μM respectively, inhibit adipocyte differentiation by downregulation of adipogenesis-related genes PPARγ, C/EBPα and SREBP-1c transcription factors and downstream genes (GLUT4, CD36 and FASN) meaning that VOO phenolics may reduce the size of fat cells and be of benefit in reducing the risk of obesity [66]. 

In an animal model of high fat diet-induced obesity, hyperglycemia, hyperlipidemia, and insulin resistance, it was shown that hydroxytyrosol decreased high fat diet-induced lipid deposits. The mechanism of action was the inhibition of the SREBP-1c/FAS pathway in liver and skeletal muscle tissues, an increase in antioxidant enzyme activities, and the normalization of the expression of mitochondrial complex subunits and mitochondrial fission marker Drp1 [80]. Further, Scoditti and colleagues [67] reported that hydroxytyrosol, at nutritionally relevant concentrations, exert significant actions, including adiponectin downregulation in inflamed adipocytes through attenuation of JNK-mediated PPARγ suppression [67]. Hydroxytyrosol was effective at concentrations as low as 1 μmol/L. This is in the range of plasma concentrations of hydroxytyrosol (0.01 to 10 μmol/L) that have been reported after dietary consumption of VOO [81], implying that the actions of hydroxytyrosol are relevant at physiological concentrations. 

These data indicate that VOO phenolics may have a protective role against excessive fat accumulation associated with systemic oxidative stress as well as exert beneficial effects against the development of metabolic syndrome. However, clinical studies are needed to support the evidence from in vitro and animal models. 

## 10. Conclusions

To conclude, the beneficial effects of VOO phenolics on health have been researched extensively, and recent research supports earlier evidence that these components exert beneficial effects on physiological processes related to health and disease. A number of studies both in vivo and in vitro demonstrate that VOO phenolic compounds beneficially alter inflammation and have beneficial effects on markers of cancer, atherosclerosis and also genes related to obesity and metabolic syndrome. The modes of action through which olive oil phenolic compounds beneficially influence health parameters detailed in this review may explain the lower incidence of chronic inflammatory diseases amongst populations residing in the Mediterranean region, who consume large volumes of VOO in their daily diets. The evidence presented in the review concludes that oleuropein, hydroxytyrosol and oleocanthal possess potent pharmacological activities in vitro and in vivo. This expanding collection of evidence would further benefit from human intervention studies with biologically relevant concentrations of these phenolic compounds. 

## Figures and Tables

**Table 1:** In vitro and in vivo studies demonstrating the effect of oleocanthal, oleuropein, and hydroxytyrosol on markers of disease.

Olive Oil Phenolic Treatment Concentration	Health/Disease Outcome	In Vitro/In Vivo Model	Key Findings	Reference
Oleocanthal (1–25 μM)	Joint degenerative disease	Murine chondrocytes. In vitro	Oleocanthal and its derivative 231 down-regulate iNOS protein expression in LPS-challenged chondrocytes reducing nitrate levels.	Iacono et al. (2010) [48]
Oleocanthal (50 μM)	Joint degenerative disease	Murine chondrocytes, Murine macrophages In vitro	Oleocanthal attenuates IL-6 and MIP- in vitro. The anti-inflammatory actions of oleocanthal in macrophages are related to the inhibition of NO production, via iNOS down regulation, and also to the decrease of relevant pro-inflammatory cytokines.	Scotece et al. (2013) [49]
Oleocanthal (10 nM)	Neurodegenerative disease	Primary hippocampal cultures In vitro	Oleocanthal alters the oligomerization state of ADDLs while protecting neurons from the synaptopathological effects of ADDLs.	Pitt et al. (2009) [50]
Oleocanthal (100 μM)	Neurodegenerative disease	Tau fibrils In vitro	Oleocanthal inhibits tau fibrillization which is a risk factor for Alzheimer’s disease	Monti et al. (2011) [51]
Oleocanthal (100 μM)	Neurodegenerative disease	Tau Fibrils In vitro	Oleocanthal prevents fibrillization of tau by locking tau into the naturally unfolded state.	Li et al. (2009) [52]
Hydroxytyrosol (1–10 μM)	Anti-inflammatory and anti-atherosclerotic activity	Human monocytic THP-1 cells In vitro	Hydroxytyrosol blunts MMP-9 release and reduces COX-2 and NF-κB activation, suggesting a vascular protective effect.	Scoditti et al. (2014) [57]
Oleuropein (200 μg/mL) and hydroxytyrosol (50 μg/mL)	Breast cancer	Human breast adenocarcinoma (MCF-7) cells In vitro	Oleuropein and hydroxytyrosol decrease cell viability and inhibit cell proliferation, and induce cell apoptosis in breast cancer MCF-7 cells.	Han et al. (2009) [58]
Oleuropein (100μM) and hydroxytyrosol (50 μM)	Breast cancer	SKBR3 BC cells. In vitro	Oleuropein and hydroxytyrosol reduce breast cancer SKBR3 cell growth through the GPER pathway.	Chimento et al. (2014) [59]
Oleocanthal (25 μM)	Breast cancer	Human breast cancer cell lines MDA-MB-231, MCF-7 and BT-474, MDA-MB-231/GFP In vitro	Oleocanthal reduce c-Met kinase activity, cell growth, migration, and invasion of breast cancer cells.	Akl et al. (2014) [60]
Oleocanthal (25, 50 μM )	Multiple myeloma	ARH-77 cells, human myeloma-derived cell line In vitro	Oleocanthal inhibits MIP-1 expression and secretion in multiple myeloma cells and inhibits cell proliferation by inducing the activation of apoptosis mechanisms and by down-regulating ERK1/2 and AKT signal transduction pathways.	Scotece et al. (2013) [61]
Oleocanthal (20 Μm)	Prostate and pancreatic cancer	PC3 (prostate), MDA-MB-231 (breast), and BxPC3 (pancreatic) cancer cells In vitro	Oleocanthal induces cancer cell death by entering the lysosome and inhibiting ASM activity, which induces lysosomal membrane permeabilization.	LeGendre et al. (2015) [62]
Oleocanthal (10 μM)	Malignant cutaneous melanoma	A375 human melanoma cell line. In vitro	Oleocanthal has selective activity for human melanoma cells versus normal dermal fibroblasts as well inhibits ERK1/2 and AKT phosphorylation and downregulation of Bcl-2 expression.	Fogli et al. (2016) [63]
Oleocanthal (10 μM)	Breast cancer	Human breast adenocarcinoma cell line MCF-7, human ductal breast epithelial tumor cell line T47D, human colorectal adenocarcinoma cell line Caco-2, human adenocarcinoma cell line and HeLa cell line. In vitro	Oleocanthal inhibits the growth of breast cancer cell lines in a dose-dependent manner. Oleocanthal treatment produces down-regulation of phosphorylated mTOR in metastatic breast cancer cell line (MDA-MB-231).	Kanfar et al. (2015) [64]
Hydroxytyrosol (25 μM)	Hyperlipidemia	C3H10 T1/2 adipocytes In vitro	Hydroxytyrosol modifies genes related with adipocyte maturation and differentiation and inhibits lipid formation.	Warnke et al. [65]
Oleuropein (150 μM) Hydroxytyrosol (300 μM)	Obesity	3T3-L1 pre-adipocytes. In vitro	Oleuropein and hydroxytyrosol act on 3T3-L1 cells to reduce 358 preadipocyte differentiation and lipid accumulation so may regulate the size of fat cells.	Drira et al. (2011) [66]
Hydroxytyrosol (1–20 μM)	Obesity	Murine 3T3-L1 adipocytes, 3T3-L1 mouse embryo fibroblasts In vitro	Hydroxytyrosol prevents adiponectin downregulation in inflamed adipocytes through an attenuation of JNK-mediated PPARγ suppression.	Scoditti et al. (2015) [67]
Oleuropein (50 μM)	Atherosclerosis and tumor invasion.	Human monocyte-like cells In vitro	Oleuropein inhibits MMP-9 expression and reduces invasiveness of tumor cells.	Dell’Agli et al. (2010) [46]
Oleocanthal (50 μM)	Neurodegenerative disease	Bend3 cells Brain microvessels of C57BL/6 mice *n* = 6 In vivo	Results from in vitro and in vivo studies demonstrate a consistent pattern of oleocanthal controlling Aβ levels and therefore reducing the risk of Alzheimer’s disease by enhancing Aβ clearance from the brain.	Abuznait et al. (2013) [53]
Hydroxytyrosol (4 mg/kg)	Atherosclerosis	New Zealand rabbits (weight 2.5–3 kg) *n* = 8 In vivo	Hydroxytyrosol improves antioxidant status and reduces the size of atherosclerotic lesions when compared with control animals suggesting that hydroxytyrosol may have cardioprotective effects	González-Santiago et al. (2006) [68]
Oleocanthal (30 μM) in vitro in vivo 5 mg/kg/d or 10 mg/kg/d for eight weeks	Hepatocellular carcinoma	HCC cell lines (Huh-7, HepG2 and HCCLM3 BALB/c mice *n* = 6 In vivo	Oleocanthal inhibits hepatocellular carcinoma tumor growth and metastasis by inactivating STAT3 both in vitro and in vivo.	Pei et al. (2016) [69]
Oleuropein (125 mg/kg of diet)	Lung metastases	MCF-7 cells xenograft growth in ovariectomised mice *n* = 20 In vivo	Oleuropein prevents both peripulmonary and parenchyma lung metastases.	Sepporta et al. (2014) [70]
Hydroxytyrosol (100, 250, 500 mg/kg)	Inflammatory swelling and hyperalgesia	Male Sprague-Dawley rats *n* = 50. In vivo	Hydroxytyrosol decreases pro-inflammatory cytokines IL-1β and TNF-α, reducing paw inflammation.	Gong et al. (2009) [55]
